# Trends in hospital admissions and prescribing due to diseases of the digestive system in England and Wales between 1999 and 2019: An ecological study

**DOI:** 10.1097/MD.0000000000037673

**Published:** 2024-04-12

**Authors:** Hassan Alwafi, Alaa Alsharif

**Affiliations:** a Department of Pharmacology and Toxicology, Faculty of Medicine, Umm Al-Qura University, Makkah, Saudi Arabia; b Department of Pharmacy Practice, College of Pharmacy, Princess Nourah bint Abdulrahman University, Riyadh, Saudi Arabia.

**Keywords:** digestive, England, hospitalization, medications, United Kingdom, Wales

## Abstract

This study aimed to investigate the trends in diseases of the digestive system hospital admissions (DDSHA) in England and Wales between (1999–2019). Secondary objectives were to investigate the type of admission and medication prescribing related to the digestive system in England. This is an ecological study using data from the Hospital Episode Statistics (HES) database and the Patient Episode Database between April 1999 and March 2019. The rate of hospital admissions with 95% confidence intervals (CIs) was calculated by dividing the number of DDSHA by the mid-year population. The trend in hospital admissions was assessed using a Poisson model. Overall, the rate of DDSHA rose by 84.2% (from 2231.27 [95% CI 2227.26–2235.28] in 1999 to 4109.33 [95% CI 4104.29–4114.38] in 2019 per 100,000 persons, trend test, *P* < .001). The most remarkable rise in hospital admission was seen in liver diseases, followed by other diseases of intestines with 1.85-fold, and 1.59-fold, respectively. Between 2004 and 2019, the overall prescribing rate for medications related to the gastrointestinal system increased by 74.6%, and stoma care related medications prescribing rate increased by 2.25-fold, followed by drugs affecting intestinal secretions and antisecretory drugs and mucosal protectants. There was an increase in hospital admission rate due to GI diseases in the United Kingdom (UK) by 84.2% from 1999 to 2019. The most remarkable rise in the rate of hospital admissions was seen in diseases of the liver and intestine.

## 1. Introduction

Diseases of the gastrointestinal (GI) system are highly prevalent worldwide, making them a substantial burden on the healthcare system.^[1]^ GI cancers were among the leading causes of death in the United Kingdom (UK) in 2019.^[2]^ In addition, a large number of GI system diseases are chronic in nature, leading to a higher risk of morbidity and a worse quality of life for patients.^[3]^ GI disorders can be life-threatening or require hospital admission, while others such as gastro-esophageal reflux disease, peptic ulcer diseases, irritable bowel diseases, anorectal disorders, and chronic liver disease are more chronic but affect the quality of life of patients, require multiple medical follow-ups, investigations, and continuous treatments, leading to a significant burden on the economic system.^[4]^

GI diseases account for approximately 8 million deaths per year worldwide, and it is estimated that around 60 to 70 million Americans are affected by GI diseases annually.^[5]^ In a recent study in the UK using a large database, Pasvol et al reported that the incidence of inflammatory bowel disease was 28.6 (28.2–28.9) per 100,000 person-years during the study period from 2000 to 2018.^[6]^ Another study in the UK estimated that the crude incidence of liver cirrhosis was around 14.55 per 100,000 person-years, increasing from 12.05 to 16.99 per 100,000 person-years from 1992 to 2001.^[7]^ The incidence of GI diseases, including nonalcoholic fatty liver disease, alcohol-related liver disease and intestinal diseases has risen significantly in the last few years; this was even highlighted in the report by the Office for National Statistics in the UK and by the Lancet Commission into liver disease.^[8,9]^

The time trend of incidence and hospital admissions of GI diseases has changed over the last 30 years.^[10]^ Previous large, population-based studies provided important time trend information regarding the epidemiology and the cost of GI diseases.^[11,12]^ However, these studies were mainly in the United States and focused on a certain disease.

In the last 30 years, multiple interventions and pharmacological treatments have changed the practice of treating GI diseases, including authorization of a new biological treatment for inflammatory diseases, improved hygiene, growing affluence, rapid urbanization, and many changes in lifestyle, especially in westernized countries with better access to healthcare services.^[10]^ These measures may have directly affected the time trend in hospital admissions for GI diseases including liver disease. However, recent studies investigating this issue are limited. Hospital admission is costly and may be preventable in some cases; therefore, estimating the trend and types of these admissions may help in identifying patterns of increase and decrease and may be an initial step in identifying factors associated with these admissions. Therefore, the primary objective of this study was to investigate the trends in hospital admissions related to diseases of the digestive system in England and Wales between 1999 and 2019. Secondary objectives were to investigate the type of admission and medication prescribing related to the digestive system in England.

## 2. Methods

### 2.1. Data sources and study population

An observational retrospective ecological design was used to analyze data from the Hospital Episode Statistics (HES) database in England and the Patient Episode Database for Wales. These 2 databases are commonly used to explore trends in hospital admissions related to different health conditions and adverse events, and they been validated and used in clinical research.^[13–23]^ The 2 databases are regularly checked to ensure their validity and accuracy.^[24]^ HES database undergoes an automated data cleaning system, and it has been tested for its internal validity.^[25]^ Data regarding hospital admission between April 1999 and March 2019 were included. HES and patient episode database for Wales databases contain hospital admission data, outpatient clinic and emergency visits for all types of diseases of the digestive system (DDS) for patients from all age categories: below 15 years, 15 to 59 years, 60 to 74 years, and 75 years and above that occur at all National Health Service trusts. Hospital admission data recorded in HES database are further classified into several types including day case admissions, elective admissions, emergency admissions and waiting list admissions. Day case admissions include Patients who are admitted electively during the day for treatment or intervention, however, they do not require overnight stay in hospital and can be discharged. waiting list admissions include patients admitted from an elective admission list, however, the hospital where the patient should be admitted were not available, and a bed could not be booked at the time. Elective admission: where the health care provider has known about at least 24 hours in advance. Emergency admission: patients admitted to hospital for emergency intervention and care. Diseases of the digestive system hospital admissions (DDSHA) were identified using the tenth version of the International Statistical Classification of Diseases system. Data for hospital admissions in England and Wales are available from the years 1999/2000 onwards. To calculate the annual hospital admission rate for DDS, we collected mid-year population data between 1999 and 2019 from the Office for National Statistics. The digestive system related medication prescription data in England and Wales were extracted from the Prescription Cost Analysis database for the available period of 2004 to 2019. This data was estimated by the national health service Prescription Services to have around 98.5% of accuracy for patients in England and 99.2% for patients in Wales.^[26]^

### 2.2. Data analysis

Annual DDSHA rates with 95% confidence intervals (CIs) were calculated using the number of hospital admissions related to DDS divided by the mid-year population of the same year. Further stratification by age and gender and by each type of DDS were also conducted. GI related medication prescription rates were calculated using the number of GI medication prescriptions divided by the total mid-year population during the same year. A chi-square test was used to assess the difference between the hospital admission rates in 1999 and 2019. The trend in hospital admissions was assessed using a Poisson model. Pearson correlation coefficient was estimated to examine the correlation between the prescribing rate and admission rate. A 2-sided *P* < .05 was considered statistically significant. All analyses were performed using SPSS version 25 (IBM Corp, Armonk, NY, USA).

### 2.3. Funding

This study is funded by Princess Nourah bint Abdulrahman University Researchers Supporting Project number (PNURSP2024R483), Princess Nourah bint Abdulrahman University, Riyadh, Saudi Arabia.

## 3. Results

**Figure 1.**
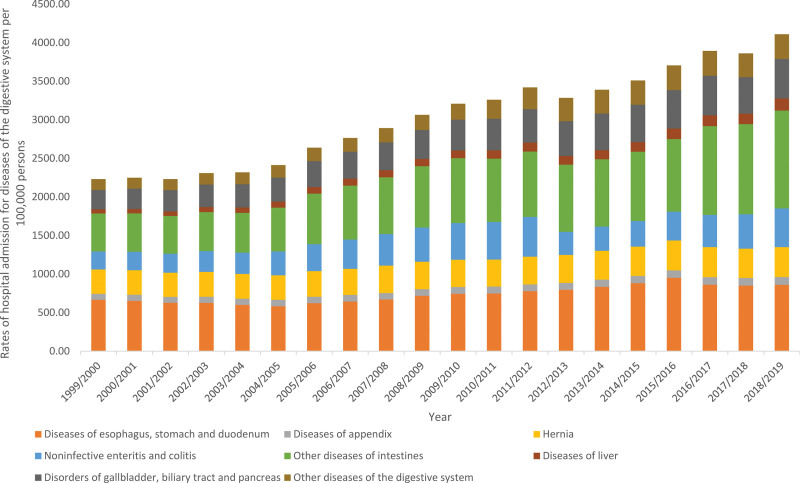
Hospital admission rates due to DDS in England and Wales stratified by type between 1999 and 2019. DDS = diseases of the digestive system.

The overall annual number for DDSHA for diverse reasons increased by 110.0% from 1163,388 in 1999 to 2442,581 in 2019, representing an increase in hospital admission rate of 84.2% (from 2231.27 [95% CI 2227.26–2235.28] in 1999 to 4109.33 [95% CI 4104.29–4114.38] in 2019 per 100,000 persons, trend test, *P* < .001) (Table 1). In the last twenty years, the most remarkable rise in the rate of hospital admission was seen in diseases of liver, followed by other diseases of intestines, other diseases of the digestive system, noninfective enteritis and colitis, and disorders of gallbladder, biliary tract and pancreas with 1.85-fold, 1.59-fold, 1.28-fold, 1.15-fold, and 1.04-fold, respectively. Furthermore, the rate of hospital admission due to diseases of appendix, diseases of esophagus, stomach and duodenum, and hernia were increased by 30.9%, 29.0%, and 22.4%, respectively (Fig. 1).

**Table 1:** Percentage of DDS admission from total number of admissions per ICD code.

ICD code	Description	Percentage from total number of admissions
K20-K31	Diseases of esophagus, stomach and duodenum (esophagitis, gastro-esophageal reflux disease, other diseases of esophagus, disorders of esophagus in diseases classified elsewhere, gastric ulcer, duodenal ulcer, peptic ulcer, site unspecified, gastrojejunal ulcer, gastritis and duodenitis, functional dyspepsia, and other diseases of stomach and duodenum)	24.2%
K35-K38	Diseases of appendix (acute appendicitis and unspecified appendicitis)	2.9%
K40-K46	Hernia (inguinal hernia, femoral hernia, umbilical hernia, ventral hernia, diaphragmatic hernia, other abdominal hernia, and unspecified abdominal hernia)	11.5%
K50-K52	Noninfective enteritis and colitis (Crohn’s disease [regional enteritis], ulcerative colitis, and other and unspecified noninfective gastroenteritis and colitis)	12.0%
K55-K64	Other diseases of intestines (vascular disorders of intestine, paralytic ileus and intestinal obstruction without hernia, diverticular disease of intestine, irritable bowel syndrome, other functional intestinal disorders, fissure and fistula of anal and rectal regions, abscess of anal and rectal regions, other diseases of anus and rectum, other diseases of intestine, and hemorrhoids and perianal venous thrombosis)	25.8%
K70-K77	Diseases of liver (alcoholic liver disease, toxic liver disease, hepatic failure, not elsewhere classified, chronic hepatitis, not elsewhere classified, fibrosis and cirrhosis of liver, other inflammatory liver diseases, and other diseases of liver)	3.3%
K80-K87	Disorders of gallbladder, biliary tract and pancreas (cholelithiasis, cholecystitis, other diseases of gallbladder, other diseases of biliary tract, acute pancreatitis, and other diseases of pancreas)	12.8%
K90-K95	Other diseases of the digestive system (intestinal malabsorption, intraoperative and postprocedural complications and disorders of digestive system, not elsewhere classified, other diseases of digestive system, complications of artificial openings of the digestive system, and complications of bariatric procedures)	7.5%

DDS = diseases of the digestive system, ICD = international statistical classification of diseases system.

### 3.1. Diseases of the digestive system hospital admissions rate by gender

**Figure 2.**
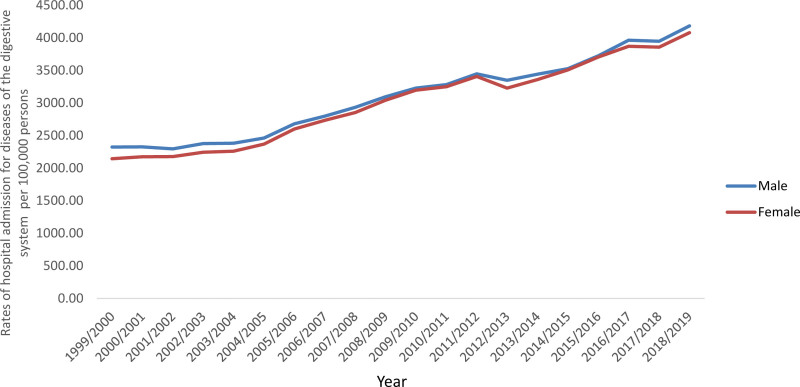
Rates of hospital admission for all DDS in England and Wales stratified by gender. DDS = diseases of the digestive system.

Females contributed to 50.1% of the total number of DDSHA accounting for 17,067,520 hospital admission episodes by a mean of 853,376 per year. DDSHA rate between females was increased by 90.2% (from 2144.43 [95% CI 2138.94–2149.93] in 1999 to 4078.94 [95% CI 4071.87–4086.01] in 2019 per 100,000 persons, trend test, *P* < .001). DDSHA rate between males was increased by 80.0% (from 2323.83 [95% CI 2317.98–2329.69] in 1999 to 4183.23 [95% CI 4175.99–4190.47] in 2019 per 100,000 persons, trend test, *P* < .001) (Fig. 2).

DDSHA rates for for diseases of esophagus, stomach and duodenum, diseases of appendix, hernia, diseases of liver, and other diseases of the digestive system were higher among males compared to females (*P* < .05), (Figure S1, Supplemental Digital Content, http://links.lww.com/MD/M30).

### 3.2. Diseases of the digestive system hospital admissions rate by age group

**Figure 3.**
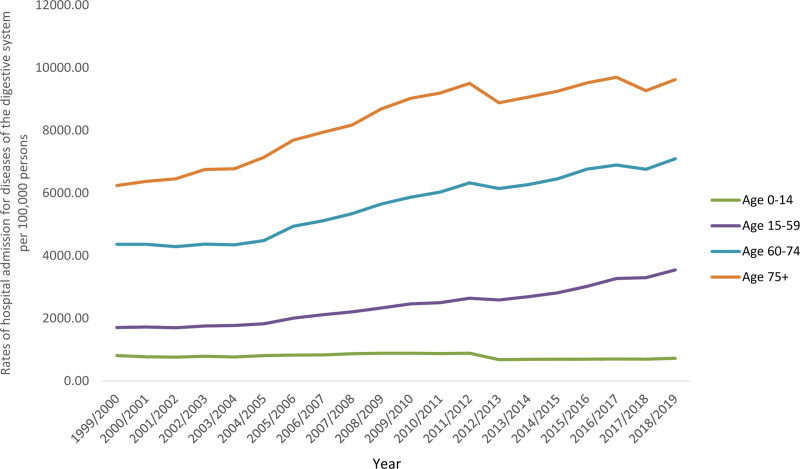
Rates of hospital admission for all DDS in England and Wales stratified by age group. DDS = diseases of the digestive system.

The age group 15 to 59 years’ accounted for 47.1% of the whole number of DDSHAs, followed the age group 60 to 74 years with 26.7%, the age group 75 years and above with 21.6%, and then the age group below 15 years with 4.6%. DDSHA rate among patients aged below 15 years increased by 10.5% (from 811.29 [95% CI 805.70–816.87] in 1999 to 726.12 [95% CI 721.04–731.20] in 2019 per 100,000 persons, trend test, *P* < .001). DDSHA rate among patients aged 15 to 59 years increased by 1.08-fold (from 1707.24 [95% CI 1702.71–1711.77] in 1999 to 3547.67 [95% CI 3541.49–3553.85] in 2019 per 100,000 persons, trend test, *P* < .001). DDSHA rate among patients aged 60 to 74 years increased by 62.5% (from 4367.14 [95% CI 4351.93–4382.35] in 1999 to 7095.64 [95% CI 7079.09–7112.19] in 2019 per 100,000 persons, trend test, *P* < .001). DDSHA rate among patients aged 75 years and above increased by 54.1% (from 6241.77 [95% CI 6217.81–6265.74] in 1999 to 9621.69 [95% CI 9596.04–9647.34] in 2019 per 100,000 persons, trend test, *P* < .001) (Fig. 3).

DDSHAs for diseases of esophagus, stomach and duodenum, hernia, noninfective enteritis and colitis, other diseases of intestines, disorders of gallbladder, biliary tract and pancreas, and other diseases of the digestive system were seen to be directly related to age. However, hospital admissions due to diseases of liver were more prevalent among the age group: 60 to 74 years, 75 years and above, 15 to 59 years, and below 15 years, respectively, (Figure S2, Supplemental Digital Content, http://links.lww.com/MD/M31).

### 3.3. Other diseases of intestines

**Figure 4.**
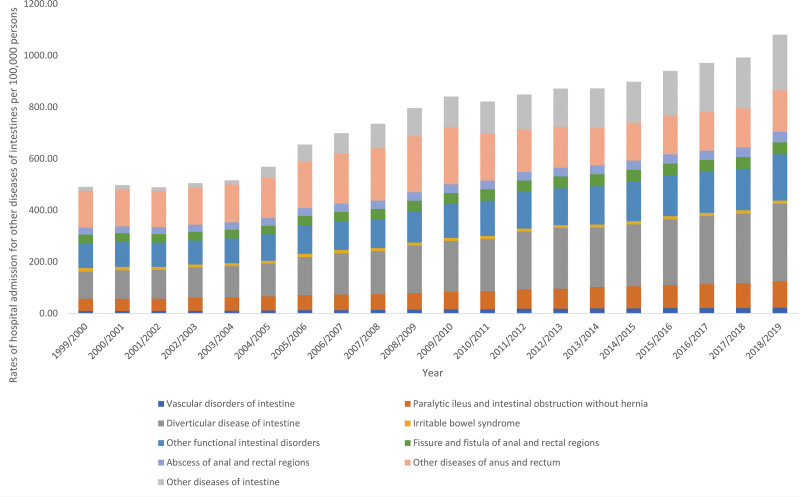
Hospital admission rates due to other diseases of intestines in England and Wales stratified by type between 1999 and 2019.

The overall annual rate for other diseases of intestines hospital admissions for diverse reasons increased by 1.59-fold (from 490.46 [95% CI 488.57–492.36] in 1999 to 1269.07 [95% CI 1266.22–1271.92] in 2019 per 100,000 persons, trend test, *P* < .05). The most increase in the hospital admission rate was seen in other diseases of intestine, diverticular disease of intestine, vascular disorders of intestine, and paralytic ileus and intestinal obstruction without hernia by 13.21-fold, 1.85-fold, 1.36-fold, and 1.15-fold, respectively (Fig. 4).

### 3.4. Diseases of liver

**Figure 5.**
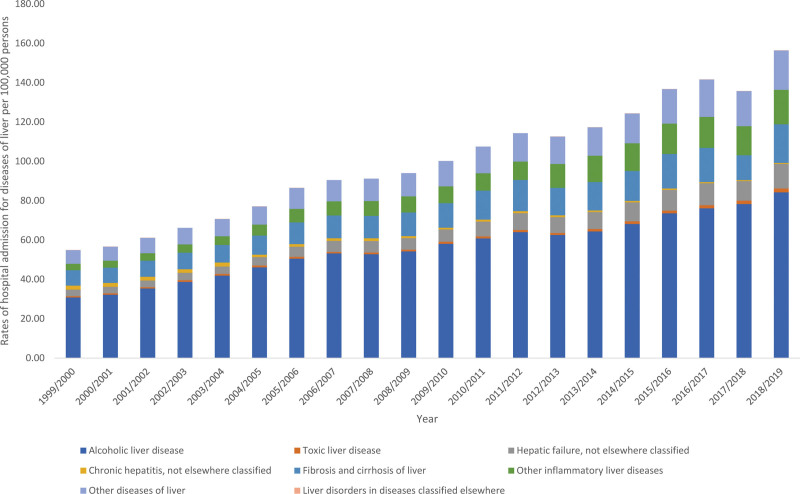
Hospital admission rates due to diseases of liver in England and Wales stratified by type between.

The overall annual rate for diseases of liver hospital admissions for diverse reasons increased by 1.85-fold (from 54.90 [95% CI 54.26–55.54] in 1999 to 156.39 [95% CI 155.39–157.40] in 2019 per 100,000 persons, trend test, *P* < .05). The most increase in the hospital admission rate was seen in liver disorders in diseases classified elsewhere, other inflammatory liver diseases, hepatic failure, not elsewhere classified, and toxic liver disease by 6.60-fold, 4.36-fold, 2.69-fold, and 2.04-fold, respectively (Fig. 5).

### 3.5. Diseases of esophagus, stomach and duodenum

**Figure 6.**
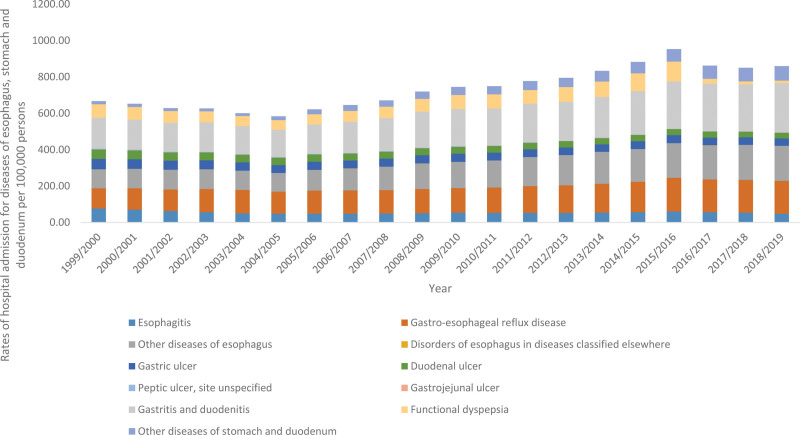
Hospital admission rates due to diseases of esophagus, stomach and duodenum in England and Wales stratified by type between 1999 and 2019.

The overall annual rate for diseases of esophagus, stomach and duodenum hospital admissions for diverse reasons increased by 29.0% (from 666.73 [95% CI 664.52–668.94] in 1999 to 860.13 [95% CI 857.78–862.47] in 2019 per 100,000 persons, trend test, *P* < .05). The most increase in the hospital admission rate was seen in other diseases of stomach and duodenum and disorders of esophagus in diseases classified elsewhere by 3.74-fold and 2.07-fold, respectively (Fig. 6).

### 3.6. Disorders of gallbladder, biliary tract and pancreas

**Figure 7.**
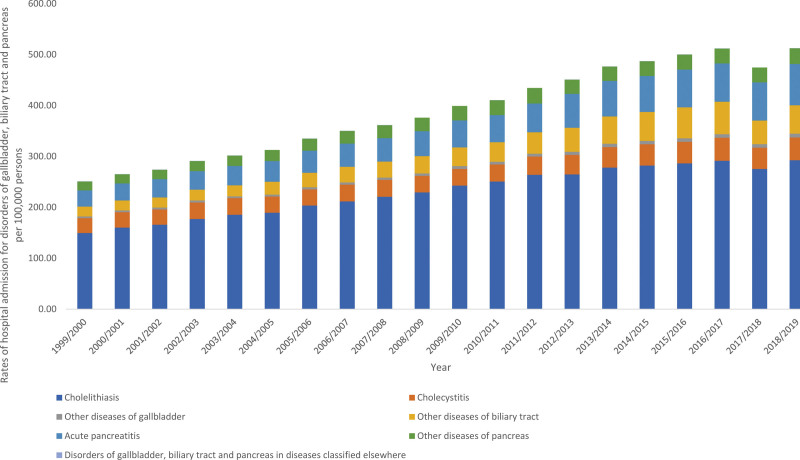
Hospital admission rates due to disorders of gallbladder, biliary tract and pancreas in England and Wales stratified by type between 1999 and 2019.

The overall annual rate for disorders of gallbladder, biliary tract and pancreas hospital admissions for diverse reasons increased by 1.04-fold (from 250.71 [95% CI 249.35–252.07] in 1999 to 512.26 [95% CI 510.45–514.08] in 2019 per 100,000 persons, trend test, *P* < .05). The most increase in the hospital admission rate was seen in other diseases of biliary tract, acute pancreatitis, and other diseases of gallbladder by 1.88-fold, 1.54-fold, and 1.23-fold, respectively (Fig. 7).

### 3.7. Other diseases of the digestive system

**Figure 8.**
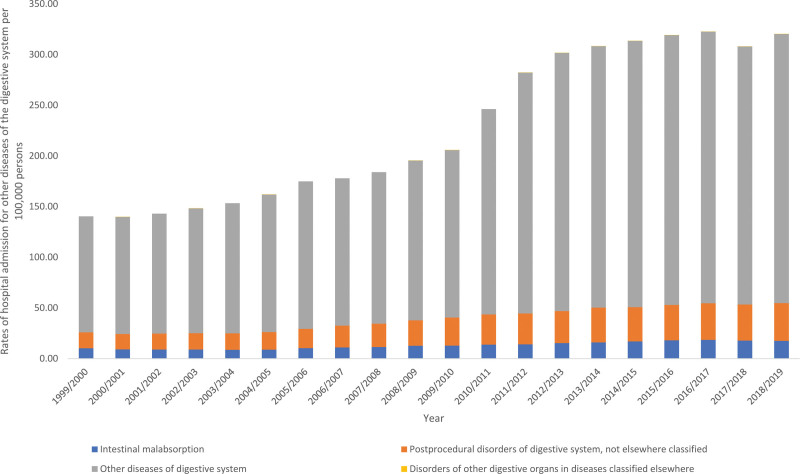
Hospital admission rates due to other diseases of the digestive system in England and Wales stratified by type between 1999 and 2019.

The overall annual rate for other diseases of the digestive system hospital admissions for diverse reasons increased by 1.28-fold (from 140.42 [95% CI 139.40–141.43] in 1999 to 320.39 [95% CI 318.96–321.83] in 2019 per 100,000 persons, trend test, *P* < .05). The most increase in the hospital admission rate was seen in postprocedural disorders of digestive system, not elsewhere classified and other diseases of digestive system by 1.37-fold, and 1.32-fold, respectively (Fig. 8).

#### 3.7.1. Hospital admission stratified by type of admission.

**Figure 9.**
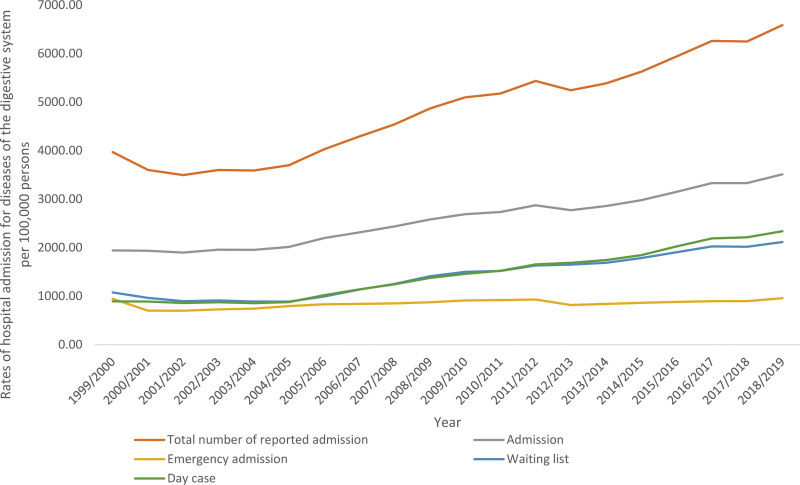
Hospital admission rates due to diseases of the digestive system in England and Wales stratified by admission type between 1999 and 2019.

Almost half of the reported number of admissions in the 2 medical databases were for regular admissions that involved inpatients treatment. Around on-third of the reported number was for elective admission (waiting list admission). The total annual rate for diseases of the digestive system admission, emergency admission, waiting list, and day case increased by 80.6%, 1.4%, 96.5%, and 1.62-fold, respectively (Fig. 9). The total annual number for diseases of the digestive system admission, emergency admission, waiting list, and day case increased by 1.06-fold, 15.6%, 1.24-fold, and 1.98-fold, respectively (Table 2).

**Table 2:** Number of reported hospital admissions stratified by type of admissions.

	Total number of reported admissions			Type of admission									Day case (elective inpatients who have been admitted for treatment just for the day)		
				Admission (involving inpatient treatment)			Emergency admission			Waiting list (indicating elective admission method)					
	England & Wales	England	Wales	England & Wales	England	Wales	England & Wales	England	Wales	England & Wales	England	Wales	England & Wales	England	Wales
1999/2000	2068,240	1985,977	82,263	1013,768	970,061	43,707	492,927	464,133	28,794	561,545	551,783	9762	465,764	462,470	3294
2000/2001	1885,855	1801,679	84,176	1013,989	969,404	44,585	367,249	338,311	28,938	504,617	493,964	10,653	466,140	462,727	3413
2001/2002	1837,694	1754,654	83,040	998,151	954,041	44,110	368,011	338,643	29,368	471,532	461,970	9562	451,620	448,061	3559
2002/2003	1901,757	1815,474	86,283	1034,730	989,165	45,565	385,340	354,514	30,826	481,687	471,795	9892	461,943	458,199	3744
2003/2004	1903,716	1816,418	87,298	1036,637	990,523	46,114	394,113	363,331	30,782	472,966	462,564	10,402	454,454	450,230	4224
2004/2005	1976,135	1885,624	90,511	1075,602	1027,632	47,970	426,019	394,730	31,289	474,514	463,262	11,252	468,868	463,674	5194
2005/2006	2164,144	2025,473	138,671	1181,192	1105,220	75,972	448,450	416,160	32,290	534,502	504,093	30,409	549,097	516,524	32,573
2006/2007	2321,608	2175,717	145,891	1251,129	1172,956	78,173	455,570	422,591	32,979	614,909	580,170	34,739	616,018	582,046	33,972
2007/2008	2474,125	2327,718	146,407	1327,206	1249,450	77,756	464,649	433,032	31,617	682,270	645,236	37,034	677,638	643,031	34,607
2008/2009	2667,644	2503,757	163,887	1414,712	1330,900	83,812	479,928	447,802	32,126	773,004	725,055	47,949	754,750	714,678	40,072
2009/2010	2816,296	2652,122	164,174	1484,801	1400,578	84,223	503,075	470,849	32,226	828,420	780,695	47,725	806,084	764,660	41,424
2010/2011	2906,572	2744,354	162,218	1536,659	1453,643	83,016	516,026	484,023	32,003	853,887	806,688	47,199	856,231	814,374	41,857
2011/2012	3075,454	2915,112	160,342	1625,090	1543,492	81,598	526,086	495,330	30,756	924,278	876,290	47,988	936,677	894,663	42,014
2012/2013	2985,459	2827,405	158,054	1579,199	1498,659	80,540	465,096	436,465	28,631	941,164	892,281	48,883	962,112	918,111	44,001
2013/2014	3092,303	2932,214	160,089	1641,307	1559,379	81,928	482,017	453,527	28,490	968,979	919,308	49,671	1001,434	956,130	45,304
2014/2015	3257,406	3097,686	159,720	1723,594	1641,962	81,632	499,428	470,290	29,138	1034,384	985,434	48,950	1069,237	1024,243	44,994
2015/2016	3469,199	3302,929	166,270	1839,954	1754,839	85,115	516,034	486,312	29,722	1113,211	1061,778	51,433	1182,672	1135,014	47,658
2016/2017	3676,466	3498,971	177,495	1957,405	1866,545	90,860	527,944	497,907	30,037	1191,117	1134,519	56,598	1286,170	1233,428	52,742
2017/2018	3692,884	3513,276	179,608	1969,264	1877,660	91,604	530,769	501,097	29,672	1192,851	1134,519	58,332	1309,448	1254,767	54,681
2018/2019	3914,376	3721,628	192,748	2086,916	1988,160	98,756	569,828	537,472	32,356	1257,632	1195,996	61,636	1390,088	1331,030	59,058

### 3.8. Gastrointestinal diseases medications prescribing

Between 2004 and 2019, the overall prescribing rate for medications related to the gastrointestinal system increased by 74.6%, stoma care related medications prescribing rate increased by 2.25-fold, followed by drugs affecting intestinal secretions and antisecretory drugs and mucosal protectants which increased by 1.84-fold and 1.39-fold, respectively (Table 3).

**Table 3:** Changes in GI medications rates from 2005 to 2019.

Prescribing rate in England and Wales									
Yr	Dyspepsia and gastro-oesophagel reflux disease	Antispasmodics and other drugs altering gut motility	Antisecretory drugs and mucosal protectants	Acute diarrhoea	Chronic bowel disorders	Laxatives	Local preparations for anal and rectal disorders	Stoma care	Drugs affecting Intestinal secretions
2005	11882.15	5778.92	49949.98	2866.95	3256.90	26169.62	3013.17	1.43	546.56
2006	11006.01	5900.41	54592.97	2894.25	3308.29	26612.51	2988.49	2.09	588.89
2007	10066.63	6168.10	59450.60	2966.08	3403.71	26822.36	2939.60	1.82	635.06
2008	9832.12	6408.66	65156.94	3058.32	3543.82	26974.38	2980.13	1.70	689.20
2009	9625.93	6592.22	71394.84	3096.60	3693.31	28010.53	3093.59	1.61	750.78
2010	9362.65	6911.50	77853.42	3160.11	3781.35	29201.98	3154.15	1.57	812.66
2011	9286.11	7240.12	83628.74	3226.39	3866.11	30138.86	3137.26	1.51	879.49
2012	9175.62	7554.15	90204.64	3285.95	3994.03	31405.47	3153.82	1.64	944.42
2013	9223.42	7909.71	97334.65	3386.84	4132.05	32461.56	3180.60	1.55	1019.29
2014	9099.41	8100.67	102306.82	3354.97	4220.68	32992.94	3049.91	2.23	1099.07
2015	9302.81	8499.86	107614.24	3408.62	4321.79	33700.51	2969.93	4.93	1194.49
2016	9343.38	8632.15	112271.72	3397.96	4379.92	33501.35	2863.54	8.31	1300.98
2017	9208.04	8686.32	115760.28	3361.39	4449.71	33812.49	2757.50	9.48	1370.76
2018	8720.47	8601.21	117659.77	3299.60	4457.77	33271.88	2562.73	6.56	1449.11
2019	8144.92	8519.56	119308.40	3192.29	4467.60	33047.68	2378.94	4.65	1553.72

GI = gastrointestinal.

#### 3.8.1. Correlation between gastrointestinal diseases medications prescribing and its associated admission rate.

When we investigated the correlation between GI diseases medications prescribing and its associated admission rate, the results showed a very strong positive correlation between GI diseases medications prescribing and its associated admission rate (r: 0.961; *P* < .001).

## 4. Discussion

The study found an increase in the overall annual number of DDSHA from 1163,388 in 1999 to 2442,581 in 2019. Previous studies investigating the trend in admissions for GI diseases in the UK are limited, and most of these studies are not specific to a certain disease. Blunt et al reported that emergency admissions in England rose by 11.8% over the 5-year period 2004/05 to 2008/09. They also reported that older people constituted the majority of these admissions.^[27]^ In the United States, similar patterns were also reported, with non-food-borne gastroenteritis, gastro-esophageal reflux disease and irritable bowel syndrome being among the most prevalent diseases.^[28]^ In addition, a study in Hong Kong analyzing data from the Hong Kong Hospital Authority found that the annual incidence of hospitalization for GI diseases increased from 4713 to 5241 per 100,000 discharges (incidence rate ratio = 1.004; 95% CI 1.003–1.005).^[10]^ They also found that GI cancers, GI infections and inflammatory diseases had the highest rate, while the incidence of peptic ulcer disease declined significantly, which is also consistent with the results reported in our study and previous literature.^[10]^

The increase in the trend of hospital admissions due to GI diseases could be attributed to multiple issues. The frailty of aging population is a major issue worldwide and is also associated with an increased risk of comorbidities and more serious diseases, which may lead to hospital admissions.^[29]^ In addition, with more medical technologies available and awareness in the community, there could be an increase in the ability to detect and treat illness.^[30]^ However, it is also important to notice that with recent developments in the medical system and the management of medical issues, better care for patients at the primary care level, better access to healthcare services, better health outcomes, a reduction in hospital admissions, more stable progression of diseases, and prevention of serious emergency issues may also be expected.^[31]^

In this study, we found that diseases of the liver had the highest increase in the trend over the 20 years of the study period, with the most increase in the hospital admission rate seen in liver disorders, alcoholic liver diseases and cirrhotic liver diseases. These results were also in line with a previous study by Thomson et al using HES data in England, in which the authors reported that admissions due to liver diseases increased during the period of 1989/1990 to 2002/2003 from 24.9 to 42.4 per 100,000 in males (71%) and from 19.3 to 27.6 per 100,000 in females (43%).^[32]^ They also reported that alcoholic liver disease was the main contributor to this increase.^[32]^ Liver diseases (alcoholic liver and cirrhosis) are major public health issues in Westernised countries.^[33]^ This was also reflected in our study, which showed that the trend in liver disease admissions was increasing in the last ten years (from 2009–2019) among males and people aged 15 to 59. When stratifying the data by gender, these results were also obvious and supported this finding, showing that men had a higher trend in liver disease admissions. The relationship between alcoholic liver and male gender has been proven previously in the literature, which supports the findings of our study.^[34,35]^ Furthermore, previous studies worldwide and in the UK have showed an increase in the trend on nonalcoholic fatty liver diseases (NAFLD).^[36–38]^ The burden of NAFLD has been a major public health concern in the last years, as the disease is associated with a higher risk of cirrhosis and hepatocellular carcinoma.^[36]^ Patients with NAFLD are also more prone to comorbidities, hospital admissions and mortality.^[39,40]^ This could also be a reason for the rise in hospital admission which was noticed in the findings of this study.

In recent years, there has been a major shift in recommendations to use biological treatments for the management of inflammatory bowel diseases, resulting in a better prognosis and fewer relapse attacks. However, our study showed that diseases of the intestine also increased in the last years. A western, sedentary lifestyle is an important risk factor linked to the development of inflammatory diseases in developed countries. In a study using HES data in England, the authors reported that age-sex standardized admission rates increased from 76.5 to 202.9 per 100,000 (*P* < .001) and from 69.5 to 149.5 per 100,000 (*P* < .001) for Crohn’s disease and ulcerative colitis, respectively, between 2003/2004 and 2012/2013. Our results were in line with these results, with similar results also reported in the United States.^[41]^

Our study also demonstrated an increase in the trend of esophageal, stomach, and duodenal diseases hospital admissions. These results could be explained by the fact that even though there has been a major reduction in the incidence of peptic ulcer diseases in England mainly due to the eradication of Helicobacter pylori,^[42]^ there has been more ingestion of nonsteroidal anti-inflammatory inhibitors, cyclooxygenase-2 inhibitors and Selective serotonin reuptake inhibitors, and cases of osteoarthritis and inflammatory diseases, which are well-known risk factors for the development of peptic ulcer diseases and dyspepsia.^[43]^ This was even highlighted in the results of this study as it showed a large proportion of the increase in the prescribing trend of GI medications was reported in the antisecretory medications.

Many factors play a vital role in the process of triggering GI tract diseases, including eating a low fiber diet, not getting enough exercise, eating large amounts of dairy products, and stress.^[44,45]^ At the same time, different methods can be implemented to decrease the risk of developing common digestive diseases, such as avoiding eating foods that contain saturated fats, following a healthy diet, exercise, not delaying defecation, and avoiding excessive intake of medicines.^[46]^

GI medications are among the most common prescribed medication in the UK, and the majority of the use of this class of medications fall into the antisecretory drugs. This was also highlighted in this study. Gastritis, peptic ulcer diseases are among the comments causes of primary care visits in the UK and this may explain this finding.

The results of this study may help to guide healthcare professionals and policy makers in understanding the trend in hospital admissions for GI diseases in the last years. The increase in the trend of hospital admissions due to certain diseases, such as liver and intestine diseases compared to other infectious diseases, such as Helicobacter pylori (H. Pylori), may reflect the actual lifestyle of the community, and it may also reflect the improvement in early identification in the healthcare system. Investigating factors associated with the increase in the rate of liver diseases and intestinal diseases is important and may help to achieve improvements in emergency department care for patients with and without GI diseases.

### 4.1. Strengths and limitations

To the best of our knowledge, this is the first study to explore trends in the rates of DDSHA in England and Wales without restricting the study to specific inclusion/exclusion criteria. Hospital admission rates for all types of DDSHA were stratified by age and gender, giving a comprehensive description of the hospitalisation profile for this group of patients over 20 years period. However, this study has some limitations. This was an ecological study, and due to the nature of data available publicly from these 2 medical databases we were unable to identify patient-level factors such as comorbidities, polypharmacy, that may impact DDSHA. In this study we did not have the capacity to explain the trends in disease prevalence and hospital admissions due to lack of population level data (which restricted our ability to adjust for important confounders) and primary care diagnosis data, however, future research using primary care data in the UK such as the Clinical Practice Research Datalink and The Health Improvement Network database may help in investigating such outcomes. Moreover, ecological studies cannot establish causality.

## 5. Conclusion

There was an increase in hospital admission rate due to GI diseases in the UK by 84.2% from 1999 to 2019. The most remarkable rise in the rate of hospital admissions was seen in diseases of the liver and intestine.

## Author contributions

**Conceptualization:** Hassan Alwafi.

**Data curation:** Hassan Alwafi.

**Formal analysis:** Hassan Alwafi.

**Investigation:** Hassan Alwafi.

**Methodology:** Hassan Alwafi, Alaa Alsharif.

**Project administration:** Hassan Alwafi.

**Resources:** Hassan Alwafi.

**Supervision:** Hassan Alwafi.

**Validation:** Hassan Alwafi.

**Visualization:** Hassan Alwafi.

**Writing – original draft:** Hassan Alwafi, Alaa Alsharif.

**Writing – review & editing:** Hassan Alwafi, Alaa Alsharif.

## Correction

The second affiliation contained a misspelling in the original article. The correct affiliation is: Department of Pharmacy Practice, College of Pharmacy, Princess Nourah bint Abdulrahman University, Riyadh, Saudi Arabia.

## Supplementary Material





## References

[1] Hellier MD Williams JG. The burden of gastrointestinal disease: implications for the provision of care in the UK. Gut. 2007;56:165–6. 17303603 10.1136/gut.2006.102889 PMC1856761

[2] World Health Organization. The top 10 causes of death. 2020. Available at: https://www.who.int/news-room/fact-sheets/detail/the-top-10-causes-of-death [access date June 10, 2023].

[3] Mokdad AA Lopez AD Shahraz S. Liver cirrhosis mortality in 187 countries between 1980 and 2010: a systematic analysis. BMC Med. 2014;12:145. 25242656 10.1186/s12916-014-0145-y PMC4169640

[4] Jeong J-J Choi M-G Cho Y-S. Chronic gastrointestinal symptoms and quality of life in the Korean population. World J Gastroenterol. 2008;14:6388–94. 19009657 10.3748/wjg.14.6388 PMC2766123

[5] Everhart JE Ruhl CE. Burden of digestive diseases in the United States part I: overall and upper gastrointestinal diseases. Gastroenterology. 2009;136:376–86. 19124023 10.1053/j.gastro.2008.12.015

[6] Pasvol TJ Horsfall L Bloom S. Incidence and prevalence of inflammatory bowel disease in UK primary care: a population-based cohort study. BMJ Open. 2020;10:e036584. 10.1136/bmjopen-2019-036584 PMC7371214 32690524

[7] Fleming KM Aithal GP Solaymani-Dodaran M. Incidence and prevalence of cirrhosis in the United Kingdom, 1992-2001: a general population-based study. J Hepatol. 2008;49:732–8. 18667256 10.1016/j.jhep.2008.05.023

[8] Public Health England. Liver disease profiles: statistical commentary, February 2020. 2020. Available at: https://www.gov.uk/government/statistics/liver-disease-profiles-february-2020-update/liver-disease-profiles-statistical-commentary-february-2020 [access date June 10, 2023].

[9] Williams R Alexander G Aspinall R. Gathering momentum for the way ahead: fifth report of the Lancet Standing Commission on Liver Disease in the UK. Lancet. 2018;392:2398–412. 30473364 10.1016/S0140-6736(18)32561-3

[10] Chan JSH Chao ACW Cheung VCH. Gastrointestinal disease burden and mortality: a public hospital-based study from 2005 to 2014. J Gastroenterol Hepatol. 2019;34:124–31. 29995979 10.1111/jgh.14377

[11] Russo MW Wei JT Thiny MT. Digestive and liver diseases statistics, 2004. Gastroenterology. 2004;126:1448–53. 15131804 10.1053/j.gastro.2004.01.025

[12] Shaheen NJ Hansen RA Morgan DR. The burden of gastrointestinal and liver diseases, 2006. Am J Gastroenterol. 2006;101:2128–38. 16848807 10.1111/j.1572-0241.2006.00723.x

[13] Zaccardi F Davies MJ Dhalwani NN. Trends in hospital admissions for hypoglycaemia in England: a retrospective, observational study. Lancet Diabetes Endocrinol. 2016;4:677–85. 27293218 10.1016/S2213-8587(16)30091-2

[14] Hunt LP Blom AW Matharu GS. The risk of developing cancer following metal-on-metal hip replacement compared with non metal-on-metal hip bearings: Findings from a prospective national registry “The National Joint Registry of England, Wales, Northern Ireland and the Isle of Man.”. PLoS One. 2018;13:e0204356. 30235326 10.1371/journal.pone.0204356 PMC6147563

[15] Naser AY Alrawashdeh HM Alwafi H. Hospital admission trends due to viral infections characterised by skin and mucous membrane lesions in the past two decades in England and Wales: an ecological study. Int J Environ Res Public Health. 2021;18:11649. 34770162 10.3390/ijerph182111649 PMC8582963

[16] Naser AY Mansour MM Alanazi AFR. Hospital admission trends due to respiratory diseases in England and Wales between 1999 and 2019: an ecologic study. BMC Pulm Med. 2021;21:356. 34749696 10.1186/s12890-021-01736-8 PMC8573565

[17] Hemmo SI Naser AY Alwafi H. Hospital admissions due to ischemic heart diseases and prescriptions of cardiovascular diseases medications in England and wales in the past two decades. Int J Environ Res Public Health. 2021;18:7041. 34280978 10.3390/ijerph18137041 PMC8297245

[18] Alwafi H. Trends in hospital admission related to poisoning by, narcotics and psychodysleptics and poisoning by antiepileptic, sedative-hypnotic, and antiparkinsonism drugs in England and Wales between April 1999 and April 2020: an ecological study. Saudi Pharma J. 2023;31:101670. 10.1016/j.jsps.2023.06.003 PMC10415227 37576854

[19] Alwafi H Naser AY Ashoor DS. Trends in hospital admissions and prescribing due to chronic obstructive pulmonary disease and asthma in England and Wales between 1999 and 2020: an ecological study. BMC Pulm Med. 2023;23:49. 36726097 10.1186/s12890-023-02342-6 PMC9893556

[20] Naser AY Dahmash EZ Al-Daghastani T. An ecological analysis of hospitalization patterns for diseases of the nervous system in England and Wales over the Last 20 Years. Healthcare (Basel, Switzerland). 2022;10:1670. 36141282 10.3390/healthcare10091670 PMC9498440

[21] Mustafa Ali S Naser AY Alghanemi AG. Musculoskeletal System and connective tissue related hospital admission in England and Wales Between 1999 and 2019: An Ecologic Study. Cureus. 2022;14:e32453. 36644035 10.7759/cureus.32453 PMC9834604

[22] Naser AY Al-Shehri H. Admissions due to perinatal respiratory and cardiovascular disorders in england. J Multidiscip Healthc. 2023;16:199–207. 36714239 10.2147/JMDH.S396406 PMC9879026

[23] Naser AY Al-Shehri H Altamimi N. Profile of hospital admissions due to preterm labor and delivery in England. Healthcare (Basel, Switzerland). 2023;11:163. 36673531 10.3390/healthcare11020163 PMC9859329

[24] Digital N. Hospital Admitted Patient Care Activity 2023. Available at: https://digital.nhs.uk/data-and-information/publications/statistical/hospital-admitted-patient-care-activity [access date March 20, 2023].

[25] Raghupathi W Raghupathi V. Big data analytics in healthcare: promise and potential. Health Inf Sci Syst. 2014;2:3. 25825667 10.1186/2047-2501-2-3 PMC4341817

[26] NHSBSA Statistics and Data Science. Prescription Cost Analysis - England 2023. Available at: https://www.nhsbsa.nhs.uk/prescription-data/dispensing-data/prescription-cost-analysis-pca-data [access date March 20, 2023].

[27] Blunt I Bardsley M Dixon J. Trends in Emergency Admissions in England 2004–2009: Is Greater Efficiency Breeding Inefficiency? London: Nuffield Trust; 2010. Available at: https://www.kingsfund.org.uk/publications/how-nhs-performing-june-2018 [access date June, 2023].

[28] Sandler RS Everhart JE Donowitz M. The burden of selected digestive diseases in the United States. Gastroenterology. 2002;122:1500–11. 11984534 10.1053/gast.2002.32978

[29] Gillam S. Rising hospital admissions. BMJ. 2010;340:c636. 20124386 10.1136/bmj.c636

[30] Hobbs R. Rising emergency admissions. BMJ. 1995;310:207–8. 7772126 10.1136/bmj.310.6974.207 PMC2548615

[31] Shi L. The impact of primary care: a focused review. Scientifica (Cairo). 2012;2012:432892. 24278694 10.6064/2012/432892 PMC3820521

[32] Thomson SJ Westlake S Rahman TM. Chronic Liver Disease—An increasing problem: a study of hospital admission and mortality rates in England, 1979–2005, with particular reference to alcoholic liver disease. Alcohol alcohol (Oxford, Oxfordshire). 2008;43:416–22. 10.1093/alcalc/agn020 18385412

[33] Blachier M Leleu H Peck-Radosavljevic M. The burden of liver disease in Europe: a review of available epidemiological data. J Hepatol. 2013;58:593–608. 23419824 10.1016/j.jhep.2012.12.005

[34] Sagnelli E Stroffolini T Sagnelli C. EPACRON study group. Gender differences in chronic liver diseases in two cohorts of 2001 and 2014 in Italy. Infection. 2018;46:93–101. 29150796 10.1007/s15010-017-1101-5

[35] Wilsnack RW Wilsnack SC Kristjanson AF. Gender and alcohol consumption: patterns from the multinational GENACIS project. Addiction. 2009;104:1487–500. 19686518 10.1111/j.1360-0443.2009.02696.x PMC2844334

[36] Estes C Anstee QM Arias-Loste MT. Modeling NAFLD disease burden in China, France, Germany, Italy, Japan, Spain, United Kingdom, and United States for the period 2016-2030. J Hepatol. 2018;69:896–904. 29886156 10.1016/j.jhep.2018.05.036

[37] Ge X Zheng L Wang M. Prevalence trends in non-alcoholic fatty liver disease at the global, regional and national levels, 1990–2017: a population-based observational study. BMJ Open. 2020;10:e036663. 10.1136/bmjopen-2019-036663 PMC7402189 32747349

[38] Younossi Z Anstee QM Marietti M. Global burden of NAFLD and NASH: trends, predictions, risk factors and prevention. Nat Rev Gastroenterol Hepatol. 2018;15:11–20. 28930295 10.1038/nrgastro.2017.109

[39] Glass LM Hunt CM Fuchs M. Comorbidities and nonalcoholic fatty liver disease: the chicken, the egg, or both? Fed Pract. 2019;36:64–71. 30867626 PMC6411365

[40] Mann JP Carter P Armstrong MJ. Hospital admission with non-alcoholic fatty liver disease is associated with increased all-cause mortality independent of cardiovascular risk factors. PLoS One. 2020;15:e0241357. 33108366 10.1371/journal.pone.0241357 PMC7591046

[41] Bewtra M Su C Lewis JD. Trends in hospitalization rates for inflammatory bowel disease in the United States. Clin Gastroenterol Hepatol. 2007;5:597–601. 17382602 10.1016/j.cgh.2007.01.015

[42] Higham J Kang JY Majeed A. Recent trends in admissions and mortality due to peptic ulcer in England: increasing frequency of haemorrhage among older subjects. Gut. 2002;50:460–4. 11889062 10.1136/gut.50.4.460 PMC1773187

[43] Williams JG Roberts SE Ali MF. Gastroenterology services in the UK. The burden of disease, and the organisation and delivery of services for gastrointestinal and liver disorders: a review of the evidence. Gut. 2007;56(Suppl 1):1–113. 10.1136/gut.2006.117598 PMC1860005 17303614

[44] Hall EH Crowe SE. Environmental and lifestyle influences on disorders of the large and small intestine: implications for treatment. Dig Dis. 2011;29:249–54. 21734392 10.1159/000323930

[45] Wen L Duffy A. Factors influencing the gut microbiota, inflammation, and type 2 diabetes. J Nutr. 2017;147:1468S–75S. 28615382 10.3945/jn.116.240754 PMC5483960

[46] Friedman G. Gastroenterology Disease and Lifestyle Medicine. In: Mechanick JI, Kushner RF, editors. Lifestyle Medicine: A Manual for Clinical Practice. Cham: Springer International Publishing; 2016. p. 333–40.

