# Supplementary material for: Trends in hospital admissions and prescribing due to diseases of the digestive system in England and Wales between 1999 and 2019: An ecological study
Source: Medicine (Baltimore). 2024 Apr 12;103(15):e37673. doi: 10.1097/MD.0000000000037673 (PMC11018217; doi:10.1097/MD.0000000000037673)
Supplement: Supplementary file 1 [file medi-103-e37673-s001.docx]

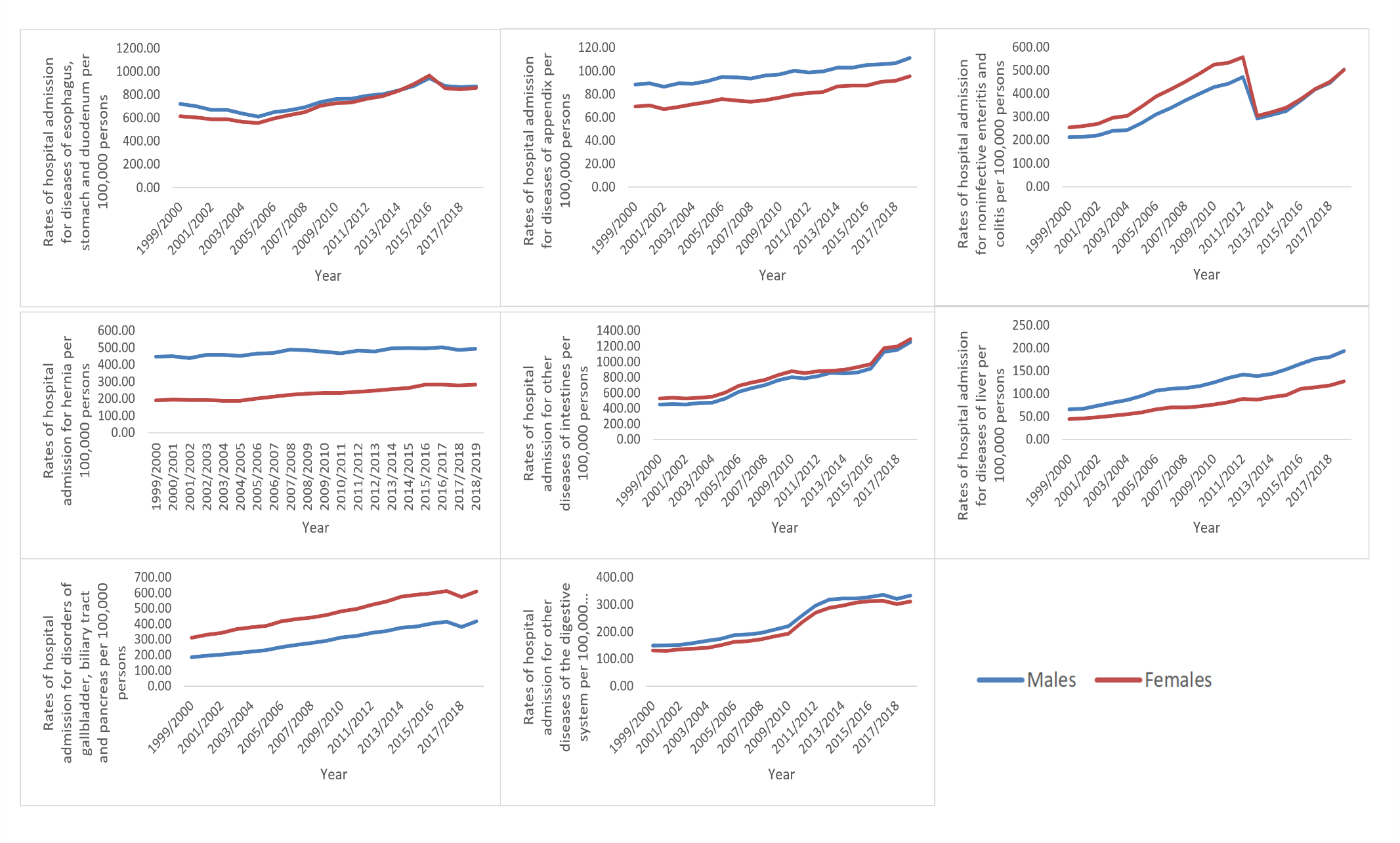


Figure S1: Hospital admission rates for diseases of the digestive system in England and Wales stratified by gender
